# Role of Muscle-Specific Histone Methyltransferase (Smyd1) in Exercise-Induced Cardioprotection against Pathological Remodeling after Myocardial Infarction

**DOI:** 10.3390/ijms21197010

**Published:** 2020-09-23

**Authors:** Qiaoqin Liang, Mengxin Cai, Jiaqi Zhang, Wei Song, Wanyu Zhu, Lei Xi, Zhenjun Tian

**Affiliations:** 1 Institute of Sports and Exercise Biology, School of Physical Education, Shaanxi Normal University, Xi’an 710119, China; liangqq@snnu.edu.cn (Q.L.); 2017mxc@snnu.edu.cn (M.C.); zhangjq1111@snnu.edu.cn (J.Z.); songd@snnu.edu.cn (W.S.); zhuwy@snnu.edu.cn (W.Z.); 2 Pauley Heart Center, Department of Internal Medicine, School of Medicine, Virginia Commonwealth University, Richmond, VA 23298-0204, USA

**Keywords:** myocardial infarction, exercise, histone methyltransferase, oxidative stress, myocardial remodeling, cardioprotection

## Abstract

Pathological remodeling is the main detrimental complication after myocardial infarction (MI). Overproduction of reactive oxygen species (ROS) in infarcted myocardium may contribute to this process. Adequate exercise training after MI may reduce oxidative stress-induced cardiac tissue damage and remodeling. SET and MYND domain containing 1 (Smyd1) is a muscle-specific histone methyltransferase which is upregulated by resistance training, may strengthen sarcomere assembly and myofiber folding, and may promote skeletal muscles growth and hypertrophy. However, it remains elusive if Smyd1 has similar functions in post-MI cardiac muscle and participates in exercise-induced cardioprotection. Accordingly, we investigated the effects of interval treadmill exercise on cardiac function, ROS generation, Smyd1 expression, and sarcomere assembly of F-actin in normal and infarcted hearts. Adult male rats were randomly divided into five groups (*n* = 10/group): control (C), exercise alone (EX), sham-operated (S), MI induced by permanent ligation of left anterior descending coronary artery (MI), and MI with interval exercise training (MI + EX). Exercise training significantly improved post-MI cardiac function and sarcomere assembly of F-actin. The cardioprotective effects were associated with increased Smyd1, Trx1, cTnI, and α-actinin expression as well as upregulated ratio of phosphorylated AMP-activated protein kinase (AMPK)/AMPK, whereas Hsp90, MuRF1, brain natriuretic peptide (BNP) expression, ROS generation, and myocardial fibrosis were attenuated. The improved post-MI cardiac function was associated with increased Smyd1 expression. In cultured H9C2 cardiomyoblasts, in vitro treatment with H_2_O_2_ (50 µmol/L) or AMP-activated protein kinase (AMPK) agonist (AICAR, 1 mmol/L) or their combination for 4 h simulated the effects of exercise on levels of ROS and Smyd1. In conclusion, we demonstrated a novel role of Smyd1 in association with post-MI exercise-induced cardioprotection. The moderate level of ROS-induced upregulation of Smyd1 may be an important target for modulating post-MI cardiac function and remodeling.

## 1. Introduction

Myocardial infarction (MI) is usually initiated by myocardial ischemia, which induces overproduction of reactive oxygen species (ROS) in the ischemic region and surrounding myocardium [1,2]. The excessive levels of ROS after MI can directly disrupt cardiac cell membranes and in turn lead to necrotic cell death in the infarcted myocardium [3]. The heart ischemic injury would further elicit pathological cardiac remodeling, which includes cardiomyocyte compensatory hypertrophy in the peri-infarcted area and cardiac fibrosis [4,5]. Although this compensatory response of cardiomyocytes may temporarily maintain cardiac contractile function, progressive cardiomyocyte hypertrophy may lead to decompensation and deterioration of cardiac function and eventually causes malignant arrhythmias and even heart failure (HF) [6]. Because of its suggested key role in mediating left ventricle (LV) remodeling after MI [3], control or reduction of oxidative stress has been a primary therapeutic target to alleviate post-MI cardiac pathological remodeling and contractile dysfunction.

SET and MYND domain containing 1 (Smyd1) is a histone methyltransferase specifically expressed in skeletal muscle and myocardium [7,8,9]. Smyd1 has been reported to play a crucial regulatory role in differentiation and maturation of skeletal and cardiac muscle cells [10,11,12], especially in sarcomere formation and assembly, muscle fibers composition, and myogenesis during cardiogenesis and ventricular development [13]; however, its role in the adult heart remains poorly understood. It was recently demonstrated that Smyd1 was significantly upregulated in a mouse model of pressure overload-induced HF and that cardiac-specific Smyd1 knockout mice developed cardiomyocyte hypertrophy that led to significant structural remodeling and severe HF [14]. On the other hand, Smyd1 was required for maintaining cardiomyocyte proliferation during the embryonic heart developmental stages and loss of Smyd1 led to lethal consequences [11]. Moreover, Franklin et al. recently suggested a role of Smyd1 as a positive regulator of cardiac metabolism and that activation of Smyd1 can prevent pathological cell growth [14]. Nevertheless, to our best knowledge, there was no previous report revealing changes and functional role of Smyd1 in post-MI heart.

ROS participates in the regulatory process of cardiomyocyte hypertrophy by activating stress-related nuclear transcription factors [15,16,17]. Thioredoxin 1 (Trx1), one of the key redox regulatory proteins, may reflect the redox state of cells and may serve as an important marker of oxidative stress [17,18]. Trx1 could also scavenge H_2_O_2_ and convert oxidative thioredoxin peroxidase into monomeric morphology, an indicator of oxidative stress-induced cellular injury [19]. It was reported that overexpression of Trx1 increased Smyd1 expression and oxidative stress tolerance in the mouse heart [18]. However, whether acute MI-induced oxidative stress significantly alters cardiac expression of Trx1 and Smyd1, which in turn modulate cardiomyocyte compensatory hypertrophy and/or pathological remodeling, remains largely unknown.

Furthermore, appropriately employed post-MI exercise training has been recommended for effective reduction of MI-associated complications and for improving cardiac function and physical activity rehabilitation in patients with MI [20]. Exercise intervention may stimulate angiogenesis in the ischemic heart, improve myocardial cell metabolism, and reduce oxidative injury caused by cardiac ischemia [21]. Exercise can also attenuate inflammation and limit scar thinning after MI [22]. Despite various modalities and mechanisms of exercise training having been studied in injured hearts [23,24,25], whether Smyd1 plays a role in cardioprotective effects of post-MI exercise training on oxidative stress and myocardial hypertrophy/remodeling in infarcted hearts remains elusive. Under this context, the purpose of our present study is to investigate the potential role of Smyd1 in post-MI ventricular remodeling and dysfunction and the potential benefits of post-MI exercise training by means of our established in vivo rat model of MI. Additional series of in vitro experiments were also conducted in H9C2 rat cardiomyoblasts using 5-amino-1-β-d-ribofuranosyl-imidazole-4-carboxamide (AICAR), a widely used activator of adenosine monophosphate (AMP)-activated protein kinase (AMPK), which is an important regulator of cell metabolism and energy sensor. The use of AICAR is to simulate a common action of various types of physical exercise, i.e., AMPK activation [26].

## 2. Results

### 2.1. Post-MI Exercise Training Enhanced Myocardial Smyd1 Expression and Reduced Oxidative Stress

**Figure 1:**
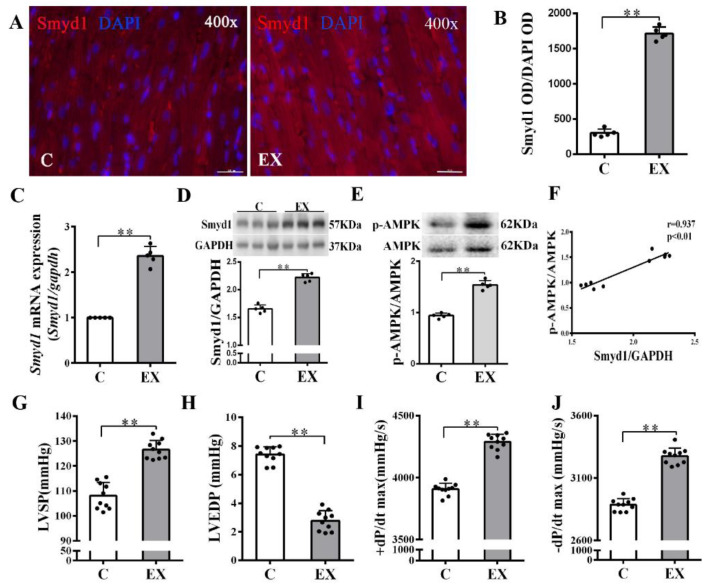
Effects of exercise training on cardiac expression of Smyd1 and cardiac function in healthy normal rats without myocardial infarction (MI): (**A**) representative immunofluorescence staining images of rat heart sections from the healthy control (**C**) and exercise (EX) groups. Smyd1 was stained in red fluorescence, and the nuclei were stained with DAPI (4′,6-diamidino-2-phenylindole) in blue color (scale bar = 100 µm); (**B**) Integrated optical density (IOD) of Smyd1 (as a ratio normalized with IOD of DAPI); (**C**,**D**) *Smyd1* mRNA levels assessed with RT-qPCR (**C**) and Smyd1 protein expression assessed with Western blots (**D**) in the heart tissues; (**E**) ratio of phosphorylated AMP-activated protein kinase (p-AMPK) and total AMPK protein expression; (**F**) correlation analysis among Smyd1 expression and the ratio of p-AMPK/AMPK; and (**G**–**J**) effects of exercise training on cardiac function parameters. Data are expressed as mean ± Standard Deviation (SD), *n* = 5/group (**B**–**E**) or *n* = 10/group (**F**–**J**). Symbols indicate ** *p* < 0.01. Abbreviations: LVSP, left ventricular systolic pressure; LVEDP, left ventricular end-diastolic pressure; and ±dP/dt_max_, the maximal rate of rise or decline of left ventricular pressure.

**Figure 2:**
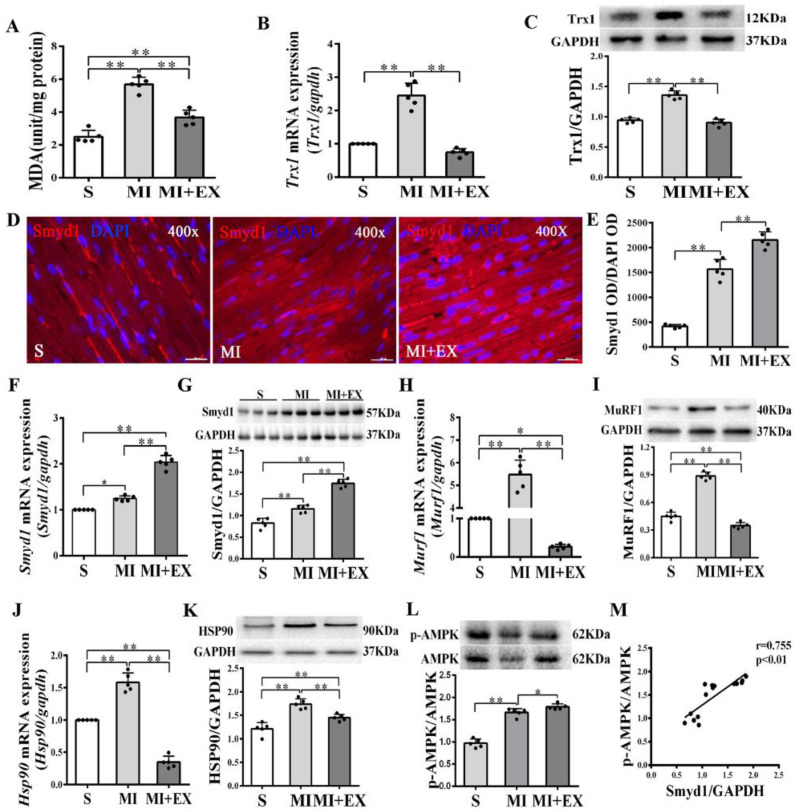
mRNA and protein expression of Smyd1 (SET and MYND domain containing 1), Trx1 (thioredoxin 1), MuRF1 (muscle RING-finger protein-1), and HSP90 (heat shock protein 90) and antioxidant capacity in the post-MI rat hearts: (**A**) Malondialdehyde (MDA) content in the infarcted myocardium; (**B**,**C**) mRNA level assessed by RT-qPCR (**B**) and protein level assessed by Western blot (**C**) of Trx1 in the infarcted myocardium; (**D**) immunofluorescent staining with Smyd1 (red color) and DAPI (blue color) in the sections from peri-infarcted area of heart (scale bar = 100 µm); (**E**) Integrated optical density (IOD) of Smyd1 (as a ratio normalized with IOD of DAPI); (**F**,**G**) *Smyd1* mRNA levels assessed with RT-qPCR (**F**) and Smyd1 protein expression assessed with Western blots (**G**) in the heart; (**H**–**K**) mRNA assessed with RT-qPCR (**H**,**J**) and protein expression assessed with Western blots (**I**,**K**) of MuRF1 and HSP90 respectively in the heart tissues; (**L**) the ratio of phosphorylated AMPK (p-AMPK) and total AMPK protein expression; and (**M**) correlation analysis among Smyd1 expression and the ratio of p-AMPK/AMPK. Data are expressed as mean ± Standard Deviation (SD), *n* = 5/group (graphs A to L, except D) or *n* = 15/group (**M**). Symbols indicate * *p* < 0.05 and ** *p* < 0.01. Abbreviations: S, Sham group; MI, MI alone group; and MI + EX, post-MI exercise training group.

In non-MI rats, exercise training significantly upregulated Smyd1 mRNA and protein expression (*p* < 0.01 group exercise alone (EX) vs. control group (C), Figure 1A–D), which was associated with improved cardiac contractile function, i.e., increase in left ventricular systolic pressure (LVSP) and ±dp/dtmax and decrease in left ventricular end-diastolic pressure (LVEDP) (*p* < 0.01, Figure 1G–J). Exercise training significantly increased the phosphorylation level of AMPK as compared with the controls (*p* < 0.01, Figure 1E), and there was a positive correlation between the ratio of p-AMPK/AMPK and the level of Smyd1 protein expression (*p* < 0.01, Figure 1F). In the hearts of the MI group, the level of oxidative stress (indicated by malondialdehyde (MDA) levels) significantly elevated along with a ROS-triggered adaptive increase in antioxidant enzyme Trx1 expression (*p* < 0.01, Figure 2A–C), as compared with the sham-operated group. Exercise training reduced MDA and Trx1 expression (*p* < 0.01) in post-MI hearts. Smyd1 mRNA and protein expression significantly increased in the MI group as compared with the sham-operated (S) group (Figure 2D–G), whereas Smyd1 expression was further upregulated in the MI with interval exercise training (MI + EX) group (*p* < 0.01 vs. the MI group, Figure 2D–G).

Furthermore, protein and mRNA expressions of Hsp90 and MuRF1, two key factors that participate in ubiquitin degradation of proteins in myocardium [27,28] significantly increased in the MI group (*p* < 0.01 vs. the S group, Figure 2H–K) and was downregulated in the MI + EX group (*p* < 0.01 vs. the MI group), indicating an inhibitory effect of exercise training on ubiquitin degradation of proteins in post-MI hearts. The phosphorylation levels of AMPK were upregulated in the MI and MI + EX groups (*p* < 0.01 or *p* < 0.05 vs. group S, respectively, Figure 2L). There was also a positive correlation between the ratio of p-AMPK/AMPK and the level of Smyd1 expression (*p* < 0.01, Figure 2M).

### 2.2. Post-MI Exercise Training Promoted Compensatory Cardiomyocyte Hypertrophy

**Figure 3:**
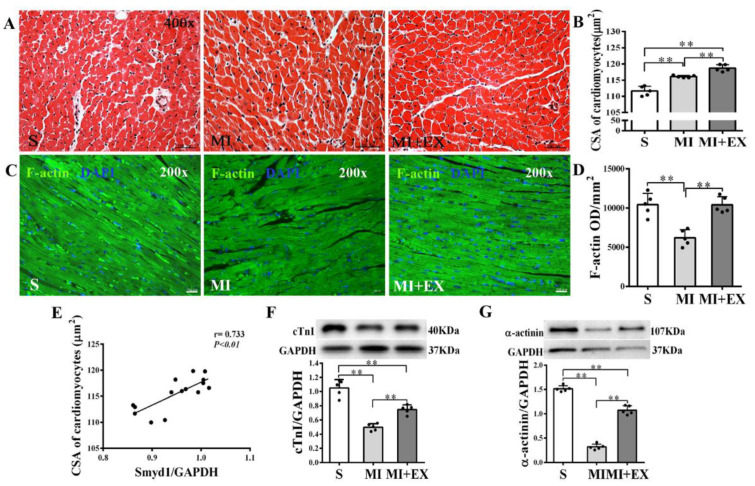
Effects of post-myocardial infarction (MI) exercise training on cardiomyocyte size in infarcted rat hearts: (**A**) Hematoxylin eosin (HE)-stained heart sections from the peri-infarcted area (scale bar = 100 µm); (**B**) quantification of the surviving cardiomyocyte cross-sectional area (CSA, *n* = 5/group); (**C**) Fluorescein isothiocyanate (FITC)-phalloidin-stained heart sections from the peri-infarcted area of myocardium (scale bar = 100 µm); (**D**) Integrated optical density (IOD) of F-actin in the peri-infarcted area of myocardium (*n* = 5/group); (**E**) correlation analysis among Smyd1 expression and CSA in cardiomyocytes (*n* = 15); and (**F**,**G**) Western blot analysis of cTnI (**F**) and α-actinin (**G**) in the infarcted myocardium (*n* = 5/group). Data are expressed as mean ± Standard Deviation (SD). Symbols indicate ** *p* < 0.01. Abbreviations: S, Sham group; MI, MI alone group; and MI + EX, post-MI exercise training group.

Cross-sectional areas (CSAs) of surviving cardiomyocytes in the peri-infarcted area were significantly enlarged in the MI group as compared with the S group (*p* < 0.01, Figure 3A,B). CSA further increased in the MI + EX group as compared with the MI group (*p* < 0.01, Figure 3A,B). These results suggested that MI triggered a compensatory hypertrophic response in the surviving cardiomyocytes, which was further enhanced by post-MI exercise training. Pearson correlation analysis revealed a significant positive correlation between CSA and Smyd1 expression (*p* < 0.01, *n* = 15, Figure 3E), suggesting a possible association of between cardiac Smyd1 expression and exercise-induced cardiomyocyte hypertrophy.

Furthermore, cardiac expressions of cTnI, α-actinin, and F-actin were significantly reduced in the MI group (*p* < 0.01 vs. the S group, Figure 3C,D,F,G) and post-MI exercise training significantly restored cTnI (*p* < 0.01 vs. the MI group, Figure 3F), α-actinin (*p* < 0.01, Figure 3G), and F-actin (*p* < 0.01, Figure 3C,D) levels in the heart. These results confirmed the difference between MI-induced hypertrophic response in surviving cardiomyocytes and exercise-induced hypertrophy, which was associated with improved myocardial cytoskeleton protein expression.

### 2.3. Smyd1 Expression and Post-MI Cardiac Function

**Figure 4:**
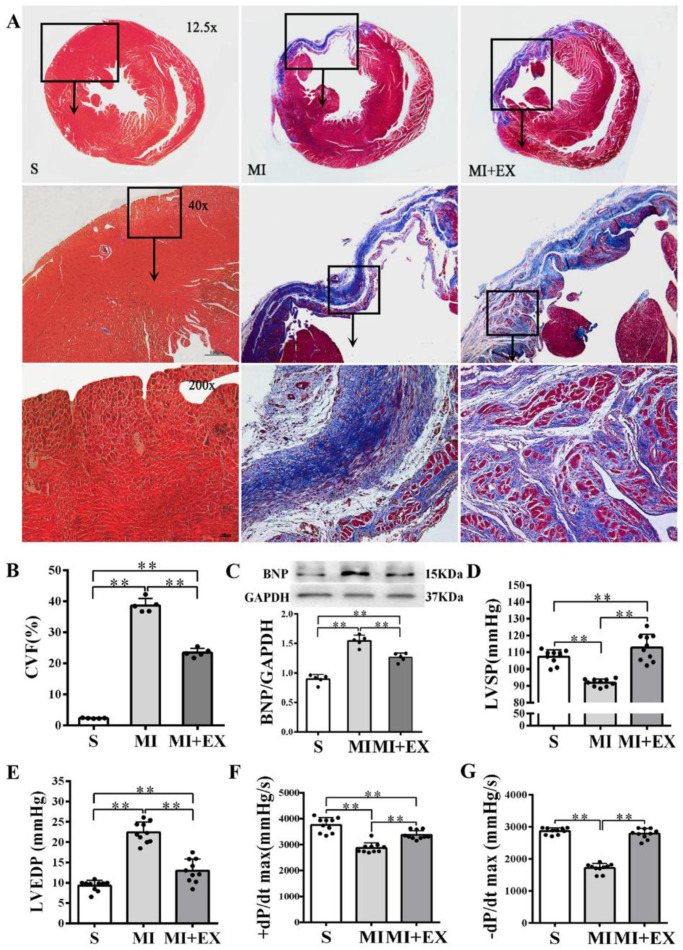
Effects of post-myocardial infarction (MI) exercise training on cardiac fibrosis and ventricular function: (**A**) Masson’s trichrome staining of cardiac tissue section for fibrosis evaluation. Images of cardiac muscle fibers (red color), collagen fibers (blue color), and nuclei (dark brown color) indicate the levels of fibrosis (scale bar = 100 µm); (**B**) quantitative analysis of collagen volume of fraction (CVF) in the post-MI rat hearts (*n* = 5/group); (**C**) Western blotting analysis of brain natriuretic peptide (BNP) expression in the infarcted myocardium (*n* = 5/group); and (**D**–**G**) effects of post-MI exercise training on cardiac function parameters (*n* = 10/group). Data are expressed as mean ± Standard Deviation (SD). Symbols indicate ** *p* < 0.01. Abbreviations: LVSP, left ventricular systolic pressure; LVEDP, left ventricular end-diastolic pressure; and ±dP/dt_max_, maximal rate of rise or decline of left ventricular pressure. Abbreviations of groups: S, Sham group; MI, MI alone group; and MI + EX, post-MI exercise training group.

MI significantly increased the collagen volume fraction (CVF) level and BNP protein expression in the heart (*p* < 0.01 vs. the S group, Figure 4A–C) and resulted in LV dysfunction manifested as significantly declined LVSP and ±dP/dtmax and elevated LVEDP (*p* < 0.01 vs. the S group, Figure 4D–G). Post-MI exercise training reduced the CVF level (*p* < 0.01, Figure 4A,B) and downregulated BNP expression (*p* < 0.05 vs. the MI group, Figure 4C). The beneficial effects of exercise training were associated with improved LV function (*p* < 0.01, Figure 4D–G).

### 2.4. Moderate Oxidative Stress Induced Cell Hypertrophy as Well as Trx1 and Smyd1 Expression in H9C2 Cardiomyoblasts In Vitro

**Figure 5:**
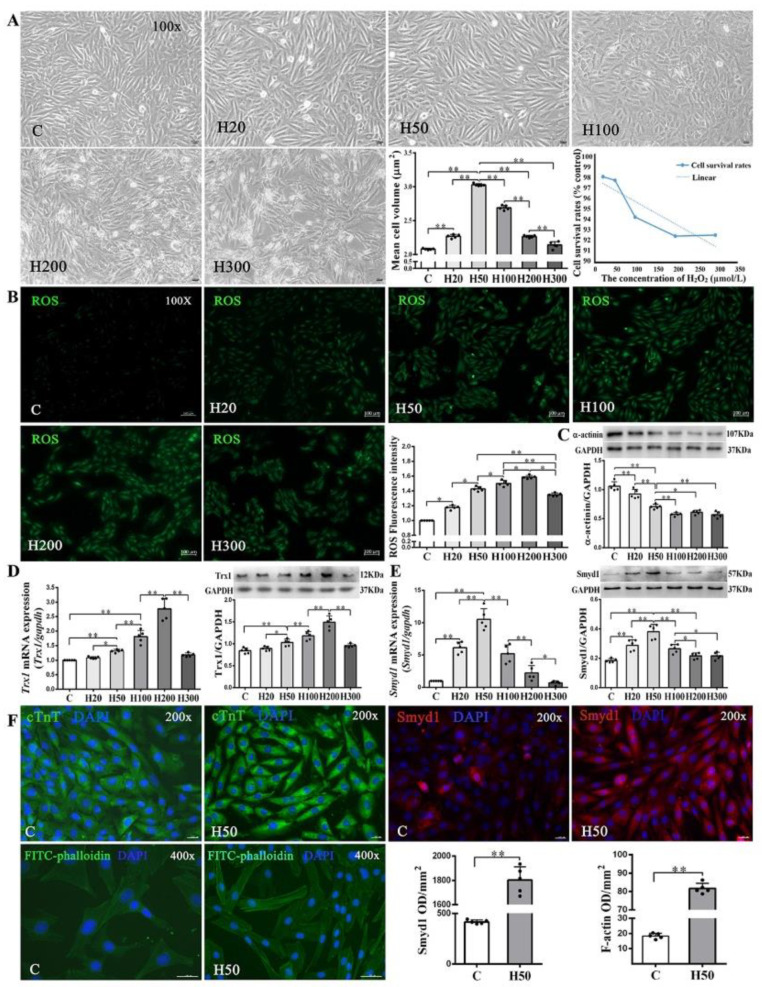
Effects of H_2_O_2_ on H9C2 cell size and Smyd1, Trx1, and F-actin expression: (**A**) cell morphology, volume, and survival rate following various concentrations of H_2_O_2_ in H9C2 cells (scale bar = 100 µm); (**B**) the level of intracellular reactive oxygen species (ROS) generation in H9C2 cells from control and H_2_O_2_ intervention groups was measured by 2′,7′-dichlorodihydrofluoresein diacetate (DCFH-DA) staining followed by quantification of fluorescence intensity; and (**C**–**E**) mRNA assessed with RT-qPCR and protein expression assessed with Western blots of α-actinin (**C**), Trx1 (**D**), and Smyd1 (**E**). Graph (**F**) shows immunofluorescence microscopy of cTnT (green color), Smyd1 (red color), and FITC-phalloidin (green color) in H9C2 cells, whereas the nuclei were stained by DAPI (blue color); scale bar = 200 µm. Data are expressed as mean ± Standard Deviation (SD), *n* = 5/group. Symbols indicate * *p* < 0.05 and ** *p* < 0.01. Abbreviations: C, Untreated control group and H20, H50, H100, H200, and H300, H9C2 cells treated with 20, 50, 100, 200, and 300 µmol/L H_2_O_2_, respectively.

Since low-concentrations of ROS induce cardiomyocyte growth [29,30], we treated H9C2 cells with various concentrations (20–300 µmol/L) of H_2_O_2_. We found a threshold concentration of 100 μmol/L H_2_O_2_ at which the cell survival rate started to decline along with abnormal morphologic changes. Cell volume, a marker of cell hypertrophy, increased significantly in all H_2_O_2_-treated groups as compared with the C group, except the H300 group. The H50 group had the largest cell volume (Figure 5A). Using 2′,7′-dichlorofluorescein diacetate (DCFH-DA) staining, we further quantitatively confirmed the dose-dependent intracellular ROS accumulation under different concentrations of H_2_O_2_ (20–300 µmol/L; Figure 5B).

As shown in Figure 5D, both protein and mRNA expressions of Trx1 were significantly elevated in the H50, H100, and H200 groups but decreased in the H300 group, as compared with the C group. Since Trx1 was reported to regulate Smyd1 expression [18] and in turn affect sarcomere organization in cardiomyocytes, we also examined mRNA and protein expressions of Smyd1 and found a significant increase of Smyd1 in the H20 and H50 groups but decreases in the H100, H200, and H300 groups (Figure 5E). Such dose-dependent effects of H_2_O_2_ were also seen in α-actinin expression (Figure 5C) and FITC-phalloidin (an indicator of F-actin level; Figure 5F) in H9C2 cells.

### 2.5. Effects of AICAR on H9C2 Cell Hypertrophy and Smyd1 Expression

**Figure 6:**
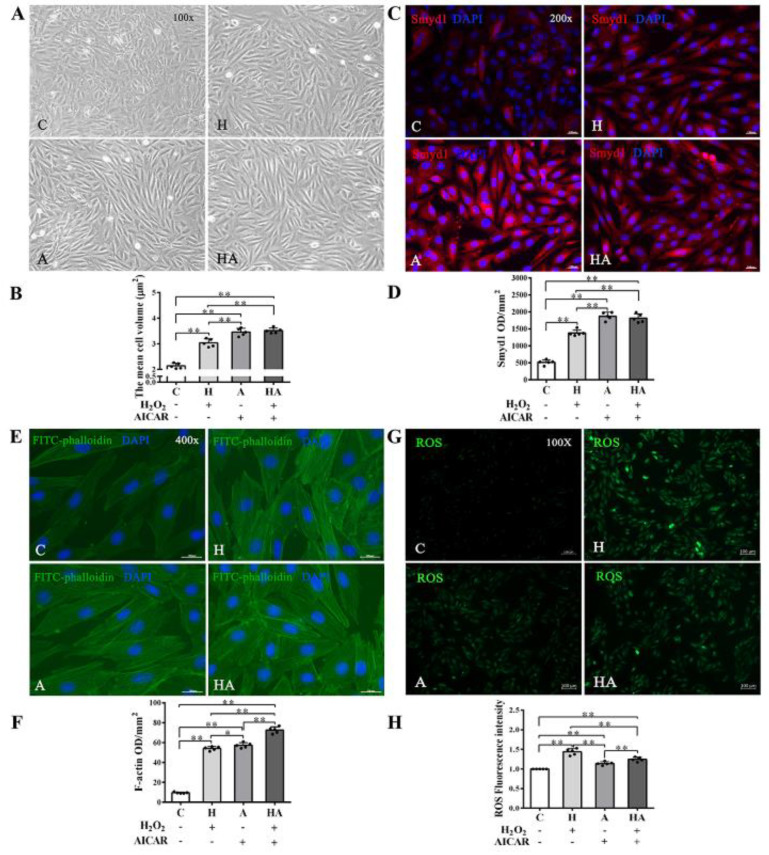
Effects of H_2_O_2_ and AMP-activated protein kinase (AMPK) agonist (AICAR) on cell volume, F-actin and reactive oxygen species (ROS) in H9C2 rat cardiomyoblasts in vitro: (**A**,**B**) cell morphology and volume of H_2_O_2_, AICAR, or H_2_O_2_ + AICAR-treated H9C2 rat cardiomyoblasts (scale bar = 100 µm); (**C**,**D**) immunofluorescence staining of Smyd1 (red color, graph **C**) and integrated optical density (IOD, graph **D**). The nuclei of cells were stained by DAPI (blue color), scale bar = 200 µm; (**E**,**F**) immunofluorescence staining of FITC-phalloidin (green color, graph **E**) and IOD in H9C2 cells (graph **F**, scale bar = 400 µm); (**G**) representative images showing ROS generation levels under various treatment conditions; and (**H**) quantified fluorescence intensity. Data are expressed as mean ± Standard Deviation (SD), *n* = 5/group. Symbols indicate * *p* < 0.05 and ** *p* < 0.01. Abbreviations: C, Untreated control group; H, H_2_O_2_-treated group; A, AICAR-treated group; and HA, H_2_O_2_ + AICAR cotreated group.

**Figure 7:**
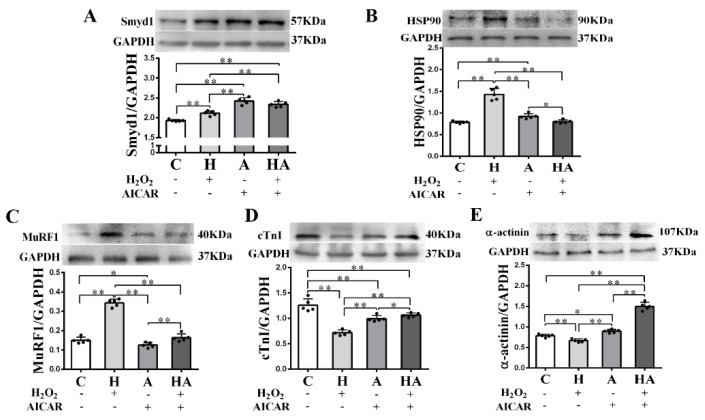
Effects of H_2_O_2_ and AICAR on expression of Smyd1 and cytoskeleton-associated proteins in H9C2 rat cardiomyoblasts in vitro: Western blot results of Smyd1 (**A**), HSP90 (**B**), MuRF1 (**C**), cTnI (**D**), and α-actinin (**E**) expressions in H9C2 cells. Data are expressed as mean ± Standard Deviation (SD), *n* = 5/group. Symbols indicate * *p* < 0.05 and ** *p* < 0.01. Abbreviations: C, Untreated control group; H, H_2_O_2_-treated group; A, AICAR-treated group; and HA, H_2_O_2_ + AICAR cotreated group.

We further investigated if treatment with AICAR (a widely used AMPK activator to simulate the key metabolic effect of exercise) would replicate the effects of exercise training in cardiac cells in vitro. We observed that low-dose H_2_O_2_ (50 µmol/L), AICAR (1 mmol/L), and H_2_O_2_ + AICAR increased cell volume (Figure 6A,B) and expression of Smyd1 (Figure 6C,D and Figure 7A) and F-actin (Figure 6E–G). AICAR or its cotreatment with H_2_O_2_ were more effective at increasing cell volume and expressions of Smyd1 and F-actin than H_2_O_2_ alone (Figure 6A–F and Figure 7A). Contrary to the H_2_O_2_-upregulated Hsp90 and MuRF1 and -downregulated cTnI and α-actinin, AICAR alone or in combination with H_2_O_2_ reduced Hsp90 and MuRF1 and increased cTnI and α-actinin (Figure 7B–E). These results indicated that AMPK activation with AICAR was more effective than low-dose H_2_O_2_ at triggering cell hypertrophic growth and suppression of protein degradation.

## 3. Discussion

The salient findings of the present study include the following: (1) Post-MI exercise training promoted physiological hypertrophy in cardiomyocyte and improved LV contractile function. (2) The cardioprotective effects of exercise training were associated with enhanced Smyd1 expression, suggesting a potential role of Smyd1 in cardioprotection against post-MI pathological remodeling and contractile dysfunction. (3) In cultured H9C2 cardiomyoblasts, moderate oxidative stress (with low-dose H_2_O_2_) or AMPK activation (with AICAR), i.e., two key contributing factors of exercise-induced cardioprotection, alone or in combination, induced cell hypertrophy and upregulated Trx1 and Smyd1 expression.

Previous studies demonstrated that MI leads to the early phase of cardiac cell necrosis/apoptosis and LV dysfunction and then the subsequent chronic phases of compensatory hypertrophy and fibrosis in survived myocardium and eventually to pathological remodeling and devastating outcomes of heart failure [3,31,32]. Protection of the survived cardiomyocytes to improve LV function in the post-MI heart have been a focal topic of cardiovascular research. Among many investigated strategies, supervised aerobic exercise has been recommended for improving cardiac function and quality of life in post-MI patients [33]. As compared with sustained low-intensity exercise, high-intensity interval exercise is more effective for improvement of aerobic capacity, anaerobic threshold, and endothelial and cardiac function in patients with heart disease [25,34]. The safety profile of post-MI exercise has been well documented as a cardiac rehabilitation modality in patients with MI or heart failure [35,36,37]. The results of our present rodent study essentially confirmed the utility of post-MI exercise training in reducing cardiac fibrosis and BNP expression, a sensitive biomarker for heart failure [38,39] and improving LV function.

Oxidative stress after MI plays a pathogenic role in post-MI cardiac remodeling [3,40]. Overproduction of ROS following MI may lead to disruption in cell membrane integrity and increased permeability that causes leakage of intracellular enzymes, and necrosis and invasion of extracellular harmful substances into intracellular space [41]. However, moderate levels of ROS participate in cell signaling transduction that regulates cell growth and survival [29,30] and a delicate balance for optimal levels of ROS is critical for maintaining cardiac health and function. The precise regulatory mechanisms involving post-MI ROS generation and myocardial remodeling remain incompletely understood.

Under this context, identification of Smyd1 as a potential participant for exercise-induced cardioprotection could be a novel and important finding. Whereas Smyd1 was mainly explored for its function in embryonic development, it was also postulated to be associated with skeletal muscle hypertrophy after exercise [7,42]. Physical stimulation such as resistance training can upregulate Smyd1, further strengthen sarcomere assembly and myofiber folding, and in turn promote physiological growth and hypertrophy of skeletal muscle [9,42,43]. Nevertheless, to our best knowledge, the present study is the first to reveal the post-MI exercise training-enhanced Smyd1 expression in myocardium and its significant positive correlation with myocardial physiological hypertrophy response and improvement of LV contractile function parameters. Smyd1 is a histone methyltransferase that participates in the regulation of myofibrillary maturation and sarcomere assembly, which are essential for muscle contraction [13]. Smyd1 regulates expression of myosin and its associated proteins such as Hsp90 and Unc-45 during myofibrils formation [7,44]. Overexpression of Smyd1 may be associated with shifts and expression of some key cardiac cytoskeletal, which ultimately translate into contractile forces in the developmental maturation of cardiomyocytes [45]. Smyd1 is also related to Trx1, a known ROS scavenger [19], because induction of cardiac expression of Smyd1 following pressure-overload via thoracic aortic constriction was more pronounced in transgenic mice with cardiac-specific overexpression of Trx1 as compared with wild-type control mice [18]. Smyd1 may also modulate endoplasmic reticulum (ER) stress response [11] and Hsp90, a molecular chaperone that is involved in Smyd1-regulated sarcomere assembly [10].

Furthermore, MuRF1, an E3 ubiquitin ligase involved in degradation of sarcomeric proteins in skeletal muscle [46], also regulates degradation of cardiac cytoskeletal proteins such as cTnI, myosin, and actin ubiquitin [47] as well as size and contractility of cardiomyocytes [28]. Our present study provided direct evidence for MI-induced cardiomyocyte compensatory hypertrophy and upregulation of MuRF1. The cell hypertrophic growth is usually associated with enhanced protein synthesis and attenuated protein degradation [48,49]. The post-MI exercise training-induced Smyd1 along with reduced MuRF1 may suggest that exercise not only triggers hypertrophic response but also inhibits pathological remodeling in infarcted hearts. Along with MuRF1, Hsp90, a molecular chaperone involved in Smyd1b-regulated sarcomere assembly [10], was upregulated following MI. Post-MI exercise training inhibited Hsp90 expression as well.

There is a consensus that exercise-induced cardioprotection is multifactorial [50]. Suppression of cellular oxidative stress under various myocardial pathologies (including MI) has been a predominantly suggested mechanism in the literature for the past 10+ years [51,52,53,54]. Our current results conceptually support the notion that exercise training enhances antioxidant defense in post-MI hearts. The reduced oxidative stress level would consequently promote cardiomyocyte cytoskeleton protein expression and sarcomere assembly in post-MI hearts.

It is notable that our abovementioned in vivo heart data were further validated in cultured cardiac cells in vitro. In H9C2 cells, we observed that mild oxidative stress with low concentrations of H_2_O_2_ (50 μmol/L) triggered cell hypertrophic growth and induced cytoskeleton proteins (cTnI and F-actin) that are important in controlling cardiomyocyte contractile function [55] and formation of myofiber and intercellular connections [56,57]. Moderate ROS also upregulated Trx1 and Smyd1 in H9C2 cells, similar to what we found in intact hearts following exercise training. Despite excessive oxidative stress after MI being a contributing factor for cardiac remodeling [3,40], due to its action to increase cell permeability and trigger cell injury signaling pathways [41], ROS may also induce cardiac angiogenesis [58] and cardioprotection [59,60] in a dose-dependent manner. Indeed, here, we found that 50-200 µmol/L H_2_O_2_ increased Trx1 expression and 300 µmol/L H_2_O_2_ led to cell death. Another interesting finding of the present study is the induction of Smyd1 by AICAR, a widely used AMPK activator and exercise memetic in H9C2 cardiomyoblasts. AICAR-induced cytoprotective effects were also superior to those afforded by low-dose H_2_O_2_, again indicating the multifactorial genesis of cytoprotection by AICAR or presumably exercise.

Taken together, our present study has focused on the novel role of Smyd1 in post-MI exercise-induced cardioprotection. Smyd1 was linked to skeletal muscle hypertrophy after exercise [43]. Stress stimulation, such as resistance training, upregulates the alternative splicing of Smyd1, further strengthens myofiber assembly and folding, and promotes physiological hypertrophic growth of skeletal muscle [9,42,43]. The association found in our present study between increased cardiac Smyd1 expression and improved LV systolic and diastolic function further suggested a similar regulatory role played by Smyd1 in both skeletal and cardiac muscles under either physiological (exercise) or pathological (MI) stressors.

Nevertheless, several limitations exist in our present study. For example, despite the in vivo and in vitro evidence provided on induction of Smyd1 by exercise, H_2_O_2_, or AICAR, we are only able to demonstrate a close association of Smyd1 induction with other cytoprotective molecules/biomarkers. Future studies are necessary to validate a causative relationship between Smyd1 and cardioprotection under post-MI exercise by means of generation and use of cardiac-specific Smyd1 knockout mice. In the present study, our assessment of LV function relied on an invasive measurement of LV pressure under surgical anesthesia at a single terminal time-point of the experiment protocol. Future studies would be ideal to combine with a noninvasive imaging approach such as rodent echocardiography, which can monitor LV contractile function decline and recovery as well as changes in ventricular wall thickness at multiple time points during the post-MI recovery period in addition to the terminal LV pressure measurements, for a more comprehensive assessment of post-MI cardiac function. Further in-depth studies are also needed to find out how Smyd1 regulates cardiomyocyte sarcomere assembly in post-MI heart. The potential interaction between Trx1 and Smyd1 is another interesting topic for more in-depth investigation. Furthermore, given the fact that cardiomyoblasts contribute to cardiac fibrosis after MI (see the review of Chen and Frangogiannis [61] for more details), further in-depth study is warranted to elucidate if induction of cell cycle arrest of cardiomyoblasts or reduction of their proliferation by post-MI ROS generation and exercise-associated oxidative stress may be a beneficial factor underlying the improvement of post-MI cardiac function by exercise training.

## 4. Materials and Methods

### 4.1. Animals

Adult male Sprague–Dawley rats (180–220 g, 3 months old) were purchased from the Laboratory Animal Centre of Xi’an Jiaotong University (Xi’an, China). The animals were housed in a temperature-controlled room (22–24 °C, humidity 50–65%) with free access to rodent chow food and water. After one week of acclimatization, rats were randomly assigned into the groups with sham or MI surgery. The animal experiment protocols were reviewed and approved by the Institutional Animal Care and Use Committee (Project ID 201916003; Approval Date: 7 July 2019) of Shaanxi Normal University (Xi’an, China). The morphometric characters of the animals are presented in Table 1.

### 4.2. In Vivo Model of Myocardial Infarction and Post-MI Intervention with Exercise Training

All in vivo animal experiments were conducted in the Laboratory of Exercise and Cardiovascular Health, Institute of Sports and Exercise Biology, Shaanxi Normal University. The rat model of MI was established by permanent ligation of the left anterior descending coronary artery (LAD) of the heart as previously described [62,63]. In brief, the rat was anaesthetized with sodium pentobarbital (30 mg/kg, i.p.) and thoracotomy was performed to expose the heart. LAD was identified approximately 2 mm under the auricle and ligated with silk suture. Regional myocardial ischemia was monitored and confirmed by electrocardiogram (ECG). Rats without ST segment elevation in their ECG were excluded due to lack of a sign of cardiac ischemia. The ECG-confirmed MI rats were then divided into two groups (*n* = 10/group): MI alone group (MI) and MI with post-MI exercise training group (MI + EX). Another Sham-operated group (S) underwent the same surgical procedure except their LAD was not ligated. Additionally, 20 non-MI rats were divided into the Control (C) and Exercise (EX) groups (*n* = 10/group).

**Figure 8:**
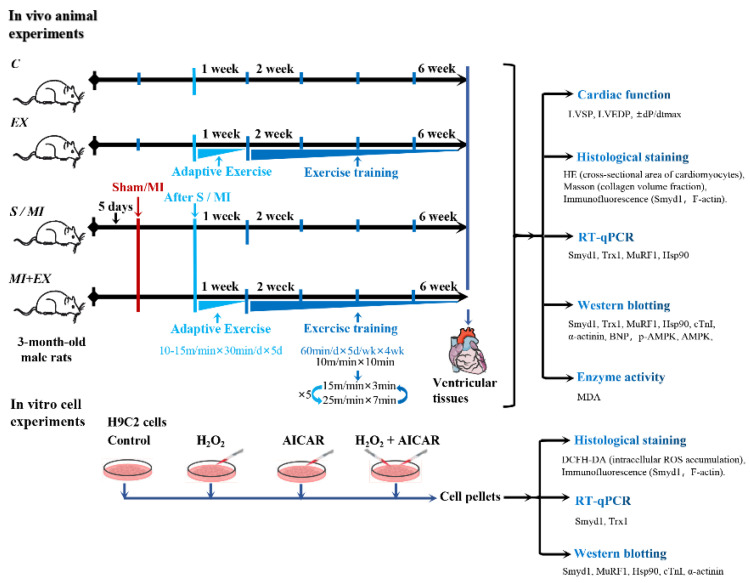
Illustrative descriptions of the experimental protocols, study methods, and investigated end-points: the in vivo experiments were performed in adult rats that underwent myocardial infarction (MI) with or without post-MI exercise training. The in vitro cellular experiments were conducted in cultured H9C2 rat cardiomyoblasts treated with or without H_2_O_2_ or exercise memetic—AICAR (5-amino-1-β-d-ribofuranosyl-imidazole-4-carboxamide). Please refer the main text for the meanings of additional abbreviations.

Both EX and MI + EX groups were subjected to interval exercise training on a motor-driven rodent treadmill as described previously [63]. As illustrated in Figure 8, the rats started with a week of adaptive exercise training at 10 m/min (40–50% VO_2max_) for 30 min. From the 2nd week, rats performed daily 50 min-sessions of interval exercise training at treadmill speeds of 10 m/min for 10 min, then 25 m/min (85–90% VO_2max_) for 7 min, and 15 m/min (50–60% VO_2max_) for 3 min. This daily exercise training went on for five days a week for a total of four weeks (Figure 8).

### 4.3. Cardiac Hemodynamic Measurement

Under surgical anesthesia, the rat was positioned in a supine position and an intraventricular catheter (polyethylene tubing #SP0109, AD Instruments, Sydney, Australia) connected to a pressure transducer (MLT1199, AD Instruments, Sydney, Australia) inserted into the right carotid artery and advanced into left ventricular (LV) cavity, from which LV systolic pressure (LVSP), LV end-diastolic pressure (LVEDP), and maximal positive and negative first derivatives of LV pressure (±dP/dtmax) were recorded and measured on a PowerLab 8/30™, ML 870 physiological polygraph system (AD Instruments). LV tissue samples were collected and immediately frozen in liquid nitrogen or fixed in 4% formaldehyde solution for further molecular biology or histological analyses.

### 4.4. Histological Staining and Analysis

The formaldehyde-fixed heart tissue samples were washed with water and underwent gradient dehydration and paraffin embedding. The paraffin-embedded heart samples were sectioned into 5 µm-thick slices and stained with hematoxylin-eosin (HE) or Masson’s trichrome to analyze cross-sectional area (CSA) of cardiomyocytes and collagen volume fraction (CVF) via computerized image analysis with Image-Pro Plus 6.0 software (IPP 6.0, IPWINMedia Cybernetics, Inc., Rockville, MD, USA). Each sample had 3 sections scanned, and 20 fields per section were viewed under microscope.

Immunofluorescence staining was performed in paraffin sections, which were deparaffinized and washed with phosphate-buffered saline (PBS). Antigen retrieval was performed with microwave oven, and then cooled down to room temperature. The slides were fixed with 4% formaldehyde solution for 60 min and washed with PBS and, after, were treated with Triton 100 for 15 min and subsequently incubated with 5% bovine serum albumin (BSA) for 30 min at 37 °C. The slides were then incubated with Smyd1 antibody (GeneTex, Irvine, CA, USA) under 1:500 dilution in paraffin sections or 1:50 dilution in cell slides overnight at 4 °C. The slides were washed with PBS the next day and incubated with horseradish peroxidase (HRP)-conjugated secondary antibody (1:1000 dilution in LV tissue sections and 1:100 dilution in cell slides, ImmunoResearch, USA) for 60 min at room temperature. The slices were washed and observed under a fluorescence microscope (Nikon Eclipse 55i, Tokyo, Japan). The nuclei were stained with DAPI (1:800 dilution, Sigma-Aldrich, St. Louis, MO, USA), and F-actin was stained with FITC-phalloidin (5 µg/mL, Sigma-Aldrich, St. Louis, MO, USA).

### 4.5. Determining MDA Levels in Heart Tissues

Frozen heart tissues (100 mg per sample) were homogenized in ice-cold PBS and centrifuged at 10,000× *g* for 10 min at 4 °C. The supernatant was recovered for measurement of malondialdehyde (MDA) levels using spectrophotometric assay kit (Jiancheng Biotech, Nanjing, China).

### 4.6. Cultured H9C2 Cardiomyoblasts

H9C2 rat cardiomyoblasts were purchased from the Cell Culture Center of the Institute of Basic Medical Sciences, Chinese Academy of Medical Sciences, Beijing, China (item No. 3111C0001CCC000219). H9C2 cells were cultured in Dulbecco’s modified eagle medium (DMEM) supplemented with 10% fetal calf serum, penicillin (100 U/mL), and streptomycin (10 µg/mL) (Gibco BRL, Grand Island, NE, USA) and were maintained at 37 °C in a 5% CO_2_ incubator (Thermo Model 371, Marietta, OH, USA). H9C2 cells were seeded in 24- or 96-well microplates, were allowed to adhere overnight, and then were treated with various concentrations of H_2_O_2_ (i.e., 0, 20, 50, 100, 200, and 300 µmol/L, labeled as groups C, H20, H50, H100, H200, and H300 respectively) for 4, 6, 12, or 24 h. Cell viability was determined by using 3-(4,5-dimethylthiazol-2-yl)-2,5-diphenyltetrazolium bromide (MTT) assay (Amresco, Solon, OH, USA), and cell morphologic changes were observed under an inverted microscope (Leica DMIL LED, Jena, Germany).

As illustrated in Figure 1, cultured H9C2 cells were randomly divided into 4 groups: control (C), H_2_O_2_ (H; 50 μmol/L, a selected dose based on the above-mentioned dose-response study), AICAR (A; 1 mmol/L), and H_2_O_2_ + AICAR (H + A). AICAR is widely used as an activator of AMPK, and it simulates AMPK-related metabolic effects of exercise at the cellular level [26].

### 4.7. Measurement of Intracellular ROS Accumulation

H9C2 cells were cultured in serum-free DMEM and incubated with 10 μmol/L 2′,7′-Dichlorofluorescein diacetate (DCFH-DA) probes (S0033, Beyotime, China) for 20 min at 37 °C in darkness, washed with serum-free DMEM 3 times, and then examined under an inverted fluorescence microscope (Zeiss Axio Observer. Z1, Jena, Germany) for detection of green fluorescence intensity that indicates ROS levels in the cells, using Image J software.

### 4.8. Determination of Cell Viability and Volume

H9C2 cells were seeded in 96-well microplates at density of 5 × 10^3^ cells per well. After treatment with the abovementioned various concentrations of H_2_O_2_ for 4 to 24 h, the cell culture media were removed and MTT solution (5 mg/mL) was added to the microplates at a final concentration of 0.05% for 1 h and replaced with formazan crystals dissolved in 200 µL dimethyl sulphoxide (DMSO; Sigma-Aldrich, St. Louis, MO, USA). The optical absorbance was measured at 490 nm wavelength using a microplate reader (BioTek Epoch, Winooski, VT, USA). Cell viability was calculated as % of control, i.e., viability (%) = (OD_490,sample_ − OD_490,blank_)/(OD_490,control_ − OD_490,blank_) × 100%. In addition, cell volume was measured in the control group and 50 μmol/L H_2_O_2_-treated group (for 4 h). Three wells were selected randomly for each group, five visual fields were selected for each well, and 10–15 cell volume in each field were measured by Image Pro-Plus (IPP) 6.0 (Media Cybernetics, Rockville, MD, USA).

### 4.9. Assessment of Gene Expression

Total RNA was extracted from frozen heart tissues (100 mg) and H9C2 cells with Trizol reagent (Invitrogen, Brazil). RNA concentration was determined spectrophotometrically at 260/280 nm wavelength using a commercial kit (BioTek Epoch, USA). First-strand cDNA was generated from RNA using Revertaid First Strand cDNA synthesis kit (TaKaRa, Kusatsu, Japan). PCR reactions were performed with quantitative PCR instrument (CFX connectTM Real-Time system, Bio-Rad, Singapore) using SYBR Green/ROX qPCR Master Mix (TaKaRa, Kusatsu, Japan) and quantified using Bio-Rad CFX manager. *Gapdh* was used as the internal control gene.

The primer sequences used for RT-qPCR analyses are shown as follows:

*smyd1*  (F: 5′-GAGGATGGTGGATGGCTACA-3′, R: 5′-TCCCGTGCCCTACTTCAAT-3′)
*trx1*  (F: 5′-CTGATCGAGAGCAAGGAA-3′, R: 5′-TCAAGGAACACCACATTGGA-3′)
*hsp90*  (F: 5′-CAATGGAGGAAGAGGAGGTC-3′, R:5′-GCGTCTGAGGAGTTGGAAAT-3′)
*murf1*  (F: 5′-GGGAACGACCGAGTTCAGACTATC-3′, R: 5′-GGCGTCAAACTTGTGGCTCA-3′)
*gapdh*  (F: 5′-CAGTGCCAGCCTCGTCTCAT-3′, R: 5′-AGGCCATCCACAGTCTTC-3′)

### 4.10. Western Blot Analysis for Protein Expression

Total protein was extracted from the heart tissues collected from the peri-infarcted area as well as H9C2 cells harvested at the end of the in vitro experiments using radio-immunoprecipitation assay (RIPA) lysate buffer (Roche, USA). The protein concentration was determined by the bicinchoninic acid protein quantitative method. The tissue/cell lysate samples were separated by 10–12% sodium dodecyl sulfate-polyacrylamide gel (SDS-PAGE) using 100 constant voltage for 1.5 h and electro-transferred (300 mA for 1.5 h at 4 °C) to nitrocellulose membranes (Millipore, USA). The membranes were incubated with 3% BSA for 60 min at room temperature followed by incubation with one of the following primary antibodies overnight at 4 °C using the following dilution concentrations and commercial suppliers: Smyd1 (1:5000; Gentex, USA); Trx1 (1:1000) and Hsp90 (heat shock protein 90; 1:500) from Cell Signaling Technology, USA; and MuRF1 (muscle RING-finger protein-1; 1:1000), brain natriuretic peptide (BNP, 1:500), α-actinin (1:2000), cardiac troponin I (cTnI, 1:500), phosphorylated AMPKα at T172 (p-AMPK, 1:1000), and AMPKα (AMPK, 1:1000), all from Abcam, USA. Glyceraldehyde-3-phosphate dehydrogenase (GAPDH, 1:5000, Bioworld, USA) was used as an internal control for equal sample loading. Membranes were subsequently washed with PBS-Tween20 (PBST) and incubated with horseradish-peroxidase (HRP)-conjugated secondary antibodies (1:5000; Jackson ImmunoResearch, West Grove, PA, USA) for 2 h at room temperature. After washing with PBST three times, the protein bands were subsequently detected with an enhanced chemiluminescence detection solution (Millipore, USA) and antibody detection was performed on a digitalized Bio-Rad ChemiDocTM MP Imaging system, Bio-Rad, USA. The results of Western blotting were analyzed using ChemiDoc MP Image Lab 5.2 (Image LabTM software, Bio-Rad, Hercules, CA, USA).

### 4.11. Statistical Analysis

Statistical analysis was performed using SPSS 17.0 statistical package. Values are presented as mean ± Standard Deviation (SD). Differences between mean values within 3 or more groups were determined by one-way analysis of variance (ANOVA). *p* < 0.05 was considered statistically significant.

## Figures and Tables

**Table 1:** Morphometric characters of the animals at pre-experiment baseline and at the end of protocol.

Group	Body Weight Basal (g)	Body Weight at End (g)	Heart Weight (mg)	Heart Rate (bpm)
C	206.9 ± 5.2	329.3 ± 7.9	1009.8 ± 20.2	427 ± 28
EX	204.9 ± 4.0	352.1 ± 14.4 ^▲▲^	1241.3 ± 17.0 ^▲▲^	412 ± 19 ^▲▲^
S	209.2 ± 3.3	325.1 ± 7.6	984.2 ± 56.3	425 ± 29
MI	212.6 ± 2.6	327.2 ± 9.6	1044.0 ± 36.7 ^●^	452 ± 26 ^●^
MI + EX	215.7 ± 4.3	340.7 ± 10.2	1209.8 ± 18.7 ^★★^	413 ± 22 ^★★^

C, Control group; EX, Exercise alone group; S, Sham-operated group; MI, Myocardial infarction group; MI + EX, MI with post-MI interval exercise training group. The values are Mean ± Standard Deviation (SD), *n* = 10/group. *Symbols*: ▲▲ *p* < 0.01 versus Group C; ● *p* < 0.05 versus Group S; ★★ *p* < 0.01 versus Group MI.

## Abbreviations

AICAR	5-amino-1-β-d-ribofuranosyl-imidazole-4-carboxamide
AMPK	Adenosine monophosphate (AMP)-activated protein kinase
ANOVA	Analysis of variance
BNP	Brain natriuretic peptide
BSA	Bovine serum albumin
CSA	Cross-sectional area
cTnI	Cardiac troponin I
CVF	Collagen volume fraction
DAPI	4′,6-Diamidino-2-phenylindole
DCFH-DA	2′,7′-Dichlorofluorescein diacetate
DMEM	Dulbecco’s modified eagle medium
DMSO	Dimethyl sulphoxide
±dP/dtmax	Maximal positive and negative first derivative of Left ventricular pressure
ECG	Electrocardiogram
ER	Endoplasmic reticulum
FITC	Fluorescein isothiocyanate
GAPDH	Glyceraldehyde-3-phosphate dehydrogenase
HE	Hematoxylin-eosin
HF	Heart failure
HRP	Horseradish-peroxidase
Hsp90	Heat shock protein 90
LAD	Left anterior descending coronary artery
LV	Left ventricle
LVEDP	Left ventricular end-diastolic pressure
LVSP	Left ventricular systolic pressure
MI	Myocardial infarction
MTT	3-(4,5-dimethylthiazol-2-yl)-2,5-diphenyltetrazolium bromide
MuRF1	Muscle RING-finger protein-1
PBS	Phosphate-buffered saline
PBST	PBS-Tween20
RIPA	Radio-immunoprecipitation assay
ROS	Reactive oxygen species
SD	Standard deviation
SDS-PAGE	Sodium dodecyl sulfate-polyacrylamide gel
Smyd1	SET and MYND domain containing 1
Trx1	Thioredoxin 1
